# Feasibility Evaluation of Preparing Asphalt Mixture with Low-Grade Aggregate, Rubber Asphalt and Desulphurization Gypsum Residues

**DOI:** 10.3390/ma11081481

**Published:** 2018-08-20

**Authors:** Xiaoliang Zhang, Ben Zhang, Huaxin Chen, Dongliang Kuang

**Affiliations:** School of Materials Science and Engineering, Chang’an University, Xi’an 710061, China; fibergroup@126.com (X.Z.); emailforpaper@sina.com (B.Z.)

**Keywords:** granite aggregate, desulphurization gypsum residues, rubber modified asphalt, asphalt mixture, pavement performance

## Abstract

Road construction consumes great amounts of high-grade natural resources. Using low-grade natural rocks or some solid wastes as substitute materials is a hot topic. Considering this, the feasibility of using low-grade granite aggregate, solid waste-based filler (desulphurization gypsum residues, DGR) and binder (waste tire rubber modified asphalt, RMA) simultaneously in asphalt mixtures has been fully investigated in this research. The commonly used base asphalt and limestone powder (LP) filler were control groups. Material characteristics of raw materials mainly including micro-morphology, functional group, mineral phase, chemical composition and thermal stability were first evaluated in order to recognize them. Four asphalt mixtures (two asphalt binder and two filler) were then designed by standard Superpave method. Finally, a detailed investigation into the pavement performance of asphalt mixtures was carried out. The moisture damage resistance and low-temperature crack resistance were detected by the changing rules of stability, strength and fracture energy, and the high-temperature stability and fatigue performance were determined by wheel tracking test and indirect tensile (IDT) fatigue test, respectively. Results suggested that RMA and DGR both showed positive effects on the low-temperature crack resistance and fatigue property of the granite asphalt mixture. DGR also strengthened moisture stability. The contribution of RMA on high-temperature deformation resistance of the granite asphalt mixture was compelling. It can offset the insufficiency in high-temperature stability made by DGR. A conclusion can be made that asphalt mixture prepared with granite, DGR and RMA possesses satisfactory pavement performances.

## 1. Introduction

In many countries, asphalt mixture is widely used in the construction of grade roads, and more than 90% of high-grade roads are paved with asphalt mixture in UK and China [1,2]. The reason why asphalt mixture is so popular is its good pavement and service performance [3,4]. In China, the road network has well developed over the past decades due to the rapid growth of the national economy. The total mileage of asphalt mixture paved expressway reached 136,500 km by the end of 2017 according to official statistics. While the road construction task is still of concern in the future because of the vast territory and national development demand.

Asphalt mixture is composed of aggregate, filler and asphalt binder [5]. The aggregate is mainly involved in the formation of the asphalt mixture skeleton. Filler and asphalt are the main components of asphalt mastic, which fills and cements the asphalt mixture skeleton [6,7]. The aggregate accounts for more than 90% of the asphalt mixture by weight. Therefore, the construction of asphalt pavement consumes great amounts of natural rocks. Many strategies have been proposed in order to reduce the supply pressure of high-quality natural aggregate. The typical method is using low-quality aggregate or some solid wastes as substitute materials [8,9,10,11,12,13,14].

The reason why some kinds of rock are known as low-quality aggregates when considering the use in asphalt mixture is that they are acidic. Gneiss, granite and quartzite are typical acidic rocks. Acidic aggregates show poor bonding performance with asphalt mastic [15]. The bonding behavior obviously affects pavement performance of asphalt mixture, especially the moisture-induced damage resistance. Many work has been done in order to improve the bonding performance of acidic aggregate-based mixture, and mainly focus on two aspects: adjusting the combination of aggregate types and modifying the asphalt mastic [8,9,16].

In terms of adjusting aggregate types, using some high-quality fine aggregate to replace the fine part of acidic aggregate-based mixture is the common technical measure. Wu et al. evaluated the performance of acidic gneiss mixture with high-quality limestone fine aggregate, which was proved to be an effective method. Chen et al. also indicated that the alkaline solid waste, steel slag, worked pretty well when used as fine aggregate in gneiss mixture [15]. The fine aggregate commonly accounts for more than 30% of asphalt mixture by volume. Using high-quality fine aggregate to replace the fine part of acidic mineral mixture is not conducive to the full use of inferior resource. Therefore, asphalt mixture with 10% of acidic aggregate is desirable.

Modifying asphalt mastic with different modifiers is a popular way to save natural resource and improve some specific performances of the asphalt mixture [9,16,17,18,19,20,21]. Previous research suggested that the use of alkaline modifiers such as cement and hydrated lime in asphalt mastic can raise the bonding performance of asphalt mixture [9,16]. While the contribution made by common alkaline filler was not as effective as high-quality fine aggregates [9]. Therefore, the key question is to prepare asphalt mastic with suitable types of filler and asphalt in order to realize the 100% use of low-grade acidic aggregate.

The desulphurization gypsum residues (DGR) are mainly generated in the power plants, which produce electricity power by burning coal. The flue gas generated during coal combustion should be purified before releasing into the air. This is because it contains some harmful ingredients such as SO_2_. Using slurry of CaCO_3_/CaO to absorb SO_2_ is a common method [22]. Finally, the generated products and excess absorbent form DGR. DGR is a strong alkali due to the excess absorbent. The alkaline feature gives it a potential to improve the bonding behavior between aggregate and asphalt mastic when used as a filler. In addition, modifying the asphalt mastic by solid waste-based modifiers is rarely reported.

Modified asphalt is considered to perform better in enhancing the performance of asphalt mixture than base asphalt binder. The amount of abandoned rubber products is increasing year by year all over the world, and waste tires form a great part of this [23]. The efficient disposal of waste rubber products is very urgent for the purpose of environmental protection and comprehensive utilization of resources. The utilization of crumb rubber for asphalt modification is proposed as a promising solution [24]. According to previous research, the rubber modified asphalt (RMA) can improve many performances of asphalt mixture such as the fatigue performance and high-temperature deformation resistance [25,26]. Considering this, the utilization of RMA in low-quality aggregate mixture may also bring benefits for the performance of asphalt mixture.

**Figure 1:**
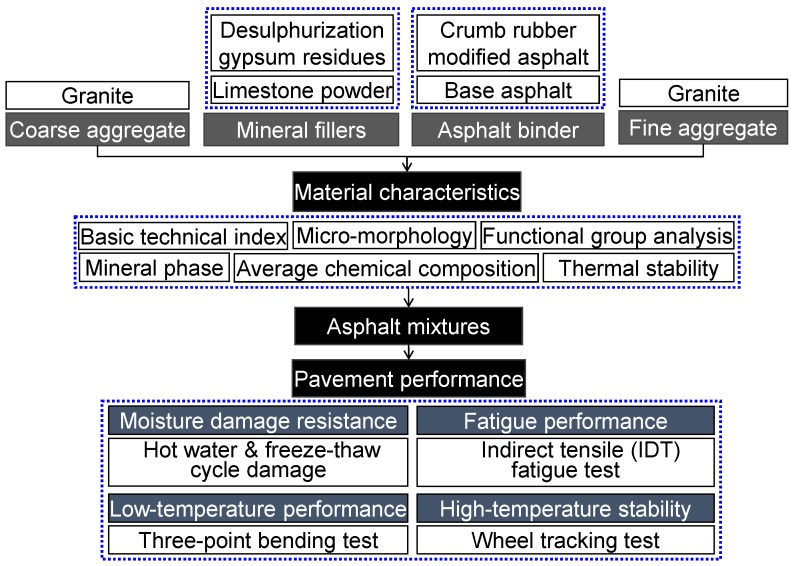
Research flow chart of this research.

Based on the above, in order to fully use the low-quality aggregate, the feasibility of preparing asphalt mixture with granite acidic aggregate, DGR and RMA were fully investigated in this research. Three studies have been conducted: (1) The basic technical indexes and main material characteristics of raw materials including micro-morphology, functional group, mineral phase, chemical composition and thermal stability were detected and compared in order to fully recognize them. (2) Designing four asphalt mixtures (two asphalt binder and two fillers) by means of the standard Superpave procedure. (3) Determining the feasibility of preparing asphalt mixture with granite aggregate, DGR and RMA by comprehensive pavement performance evaluation, including moisture damage resistance, low-temperature crack resistance, high-temperature stability and fatigue performance. The research flow chart is shown in Figure 1.

## 2. Materials and Research Methodologies

### 2.1. Materials

In this research, the coarse aggregates and fine aggregates were both granite. Limestone powder (LP) and desulphurization gypsum residues (DGR) were used as fillers. Granite aggregate and LP were provided by the Xiangyu Mining Co., Ltd., Anhui, China. DGR was from the Datang Shaanxi Power Generation Co., Ltd., Shaanxi, China. In addition, two types of asphalt binder namely base asphalt and tire rubber modified asphalt (RMA) were adopted. The base asphalt grade 80/100 was produced by the Jiangsu Baoli Asphalt Co., Ltd., Jiangsu, China. RMA was prepared in the laboratory. Crumb rubber was obtained by grinding waste tire and the maximum size of rubber particle was 60 mesh. The dosage of rubber powder was set to 20% of base asphalt grade 60/80 by weight according to the suggestion given in previous research [27,28]. The wet process was used to prepare RMA. Crumb rubber mixed with base asphalt at 185 °C, and the mixture was sheared for 90 min with a shearing rate of 4000 r/min.

### 2.2. Research Methodologies

#### 2.2.1. Technical Indexes and Material Characteristics of Raw Materials

Almost every country will establish corresponding technical specifications for road construction. There are strict requirements for the basic technical indexes of raw materials in technical specifications. Therefore, checking the basic technical indexes is the first step to determine the feasibility of raw materials. In this research, basic technical indexes of aggregate, filler and asphalt binder (density, water absorption, penetration, ductility, etc.) were tested according to Chinese standard methods [29,30].

Except for the basic technical indexes, the material characteristics of raw materials also directly affect the performance of asphalt mixture. Hence, the main material characteristics of raw materials were further detected in order to fully understand them, especially for the uncommonly used DGR and RMA. The micro-morphology of aggregate, filler and tire rubber powder was observed by the Scanning Electron Microscope (SEM, Hitachi, Japan). The functional groups of base asphalt and RMA were determined by the Fourier Transform Infra-red Spectrometer (FT-IR, Thermo Fisher, Waltham, MA, USA). The X-ray Diffraction (XRD, Bruker, Karlsruhe, Germany), and Polarizing Optical Microscopy (POM, Olympus Corporation, Tokyo, Japan), X-ray Fluorescence (XRF, Panalytical Axios, Almelo, The Netherlands), Thermal Gravimetric (TG, Netzsch, Selb, Germany) analysis system were used to characterize the mineral phase, average chemical composition, thermal stability of aggregate and filler, respectively.

#### 2.2.2. Design of Asphalt Mixtures

Four asphalt mixtures with a nominal maximum size of 12.5 mm were designed by Superpave method. The combination of raw material types for each mixture is shown in Table 1. The only difference for these four mineral mixtures is the types of filler. So the four mixtures can be divided into two categories when designing the hybrid gradation of mineral materials (aggregate and filler), namely mixture of granite and LP (M_GL_) and mixture of granite and DGR (M_GD_).

**Figure 2:**
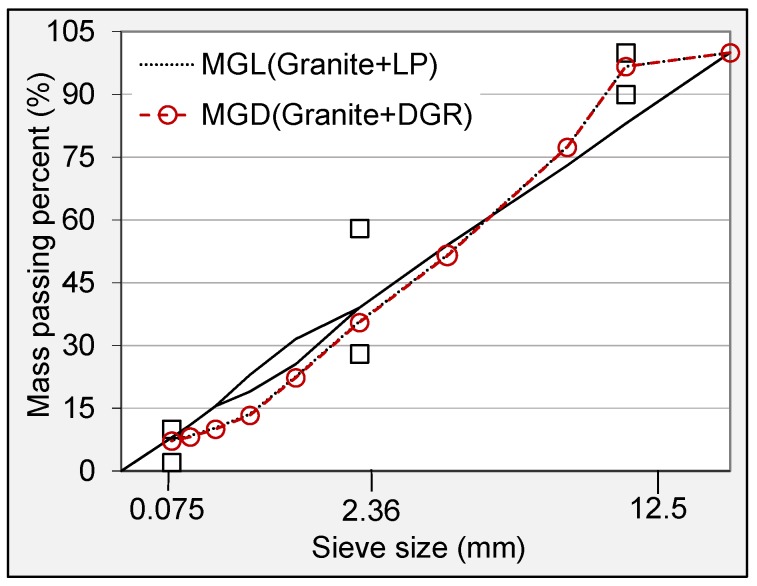
Hybrid gradations used in this research.

In order to keep volume compositions of all mineral mixtures simultaneous, the same blending proportion of raw materials for each mixture was used. Coarse aggregate, fine aggregate and filler accounted for 63%, 32% and 5% by total volume of hybrid mineral mixture, respectively. The hybrid gradations used in this research are shown in Figure 2. The optimum asphalt content (OAC) and volumetric property indexes for each asphalt mixture were determined and checked according to the Superpave design procedure.

#### 2.2.3. Performance Evaluation of Asphalt Mixtures

##### Moisture-Induced Damage Resistance

The moisture stability of asphalt mixtures was first investigated by the hot water damage test and freeze–thaw damage test, and then further analyzed by means of the energy method. The tested samples were prepared by coring and cutting the cylindrical specimens compacted in Superpave gyratory compactor, and the final dimension of prepared samples was 50 mm thickness, 100 mm in diameter. The air voids of samples for hot water damage test and freeze–thaw damage test were 4–6% and 6–8%, respectively. A total of 12 samples for each asphalt mixture were prepared. They were equally divided into four groups; one was the control group, the others were conditioned groups.

Hot water damage test and freeze–thaw damage test were conducted according to the Chinese standard methods [29]. For hot water damage test, the control group and three conditioned groups were first immersed in water bath of 60 °C for 0.5, 24, 48 and 72 h, respectively. The Marshall stabilities of all samples were then determined by the Marshall stability tester. The parameter of retained Marshall stability (RMS), reflecting hot water damage resistance of asphalt mixture, was computed according to Equation (1).

(1) $$\mathrm{RMS}=\frac{{\mathrm{MS}}_{n}}{{\mathrm{MS}}_{0}}\times 100\%$$

where MS_0_ is the average Marshall stability of control group, and MS*_n_* is the average stability of conditioned group (*n* = 24, 48 and 72 h).

For freeze–thaw damage test, three conditioned groups were subjected to freeze–thaw damage for 1, 2 and 3 cycles, respectively. A single freeze–thaw damage process was composed of freezing at −18 °C for 16 h and thawing at 60 °C for 24 h. All samples of control and conditioned groups were moved to water bath of 25 °C for 2 h to keep the temperature same before being tested. The splitting failure of all samples was conducted by the splitting test instrument with real-time data collection module. The parameter of tensile strength ratio (TSR), reflecting freeze–thaw damage resistance of asphalt mixture, was computed according to Equation (2).

(2) $$\mathrm{TSR}=\frac{{\mathrm{SS}}_{i}}{{\mathrm{SS}}_{0}}\times 100\%$$

where SS_0_ is the average splitting strength of control group, and SS*_i_* is the average splitting strength of conditioned group (*i* = 1, 2 and 3 cycles).

**Figure 3:**
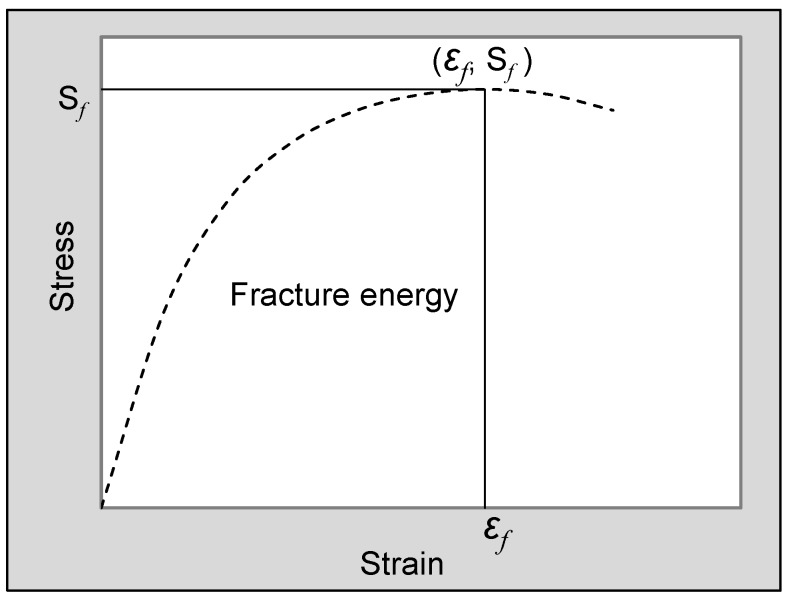
Determination mechanism of fracture energy.

Except for the traditional parameters (RMS and TSR), the change rule of energy parameter was also commonly adopted in moisture stability evaluation [8]. Fracture energy (E*_f_*) can be determined based on the stress–strain curve drawn according to the splitting test raw data as shown in Figure 3, and computed according to Equation (3). The energy parameter of retained energy ratio (RER) under different freeze–thaw damage cycles for each asphalt mixture was determined by Equation (4). The winner of four asphalt mixtures in freeze–thaw damage resistance can be decided by comparing their RER values. The energy method involved the strain and stress of sample simultaneously. It has been reported to be more scientific than traditional RMS and TSR methods, which only consider the change of single stability and strength (stress).

(3) $$E_{f}=\int_{0}^{\varepsilon_{f}}S\left(\varepsilon \right)d\varepsilon$$

where ε*_f_* is the failure strain of sample, at which the splitting strength is also largest (S*_f_*), and S(ε) is the real-time splitting strength of sample when strain is ε.

(4) $$\mathrm{RER}=\frac{E_{fi}}{E_{f0}}\times 100\%$$

where E*_fi_* is the average fracture energy of samples in conditioned group with freeze–thaw damage for *i* cycles, and E*_f_*_0_ is the average fracture energy of samples in control group.

##### Low-Temperature Crack Resistance

The three-point bending test, conducted according to the Chinese standard method [29], was adopted to evaluate the low-temperature performance of asphalt mixtures. The used beam samples with a dimension of 250 mm length, 30 mm width and 35 mm height were processed by cutting roller compacted large-size concrete slabs. Three beam samples for each mixture were prepared. The test was conducted by a universal test machine (UTM) at −10 °C, forcing the beam sample into bending and fracture with a deformation rate of 50 mm/min. Samples were placed in a temperature-controlled chamber for 4–5 h in advance to maintain the surface and internal of sample at the same test temperature. The real-time strain and stress (flexural strength) of the tested sample were obtained based on previously obtained test data. Fracture energy was also determined according to Equation (3). The low-temperature crack resistance ability of asphalt mixture was assessed by the failure strain and fracture energy.

##### High-Temperature Deformation Resistance

The wheel tracking test, also conducted according to the Chinese standard method [29], was adopted to reveal the high-temperature stability of asphalt mixture. The used slab samples, prepared by a roller compaction, were 300 mm length, 300 mm width and 50 mm height. Three replicates for each mixture were considered. Samples were placed in a wheel tracking device to raise the temperature to 60 °C beforehand. The test was conducted under repeated wheel loading. For the standard wheel tracking test, the wheel pressure was 0.7 MPa. In order to reveal the high-temperature behavior of asphalt mixture under overloading, 1.0 MPa was also selected. The test process was simple; the wheel rolled back and forth on the surface of samples along the axis of symmetry with a wheel speed of 42 pass/min for 1 h, and the real-time rutting depth was recorded. The dynamic stability (DS), reflecting the high-temperature stability of asphalt mixture, was computed according to Equation (5).

(5) $$\mathrm{DS}=\frac{15\times 42}{d_{60}-d_{45}}$$

where *d*_45_ and *d*_60_ are rutting depths of slab samples when testing time reaches 45 min and 60 min respectively, mm.

##### Fatigue Crack Resistance

**Figure 4:**
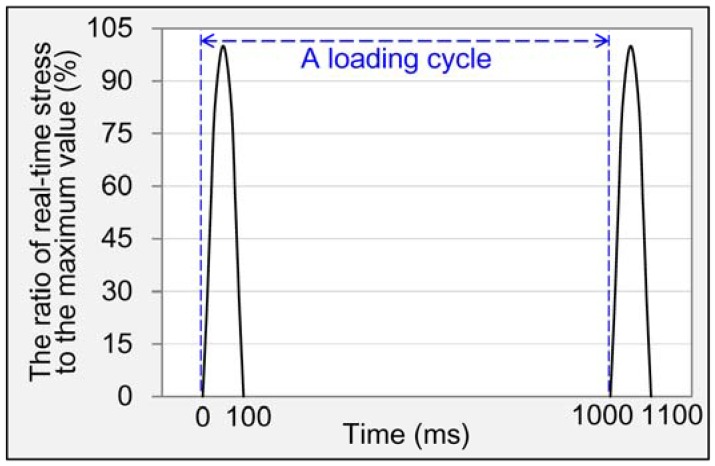
Loading signal used in this research.

The fatigue crack resistance of asphalt mixture was evaluated by means of indirect tensile (IDT) fatigue test according to AASHTO-TP31 standard [31]. The samples were the same as those used in hot water damage test. IDT fatigue test was also conducted by UTM. The test temperature was 15 °C, and samples were moved to a temperature control chamber of UTM to keep the surface and internal parts at the same test temperature. Stress-controlled mode with three stress levels of 400, 500 and 600 kPa was adopted, and loading signal was haversine waveform as shown in Figure 4. Three replicates for every asphalt mixture at each stress level were considered. The loading number, at which the main radial crack propagated completely through the specimen, was defined as fatigue life (*N_f_*).

## 3. Results and Discussions

### 3.1. Technical Properties and Material Characteristics of Raw Materials

#### 3.1.1. Technical Properties

The basic technical properties of raw materials should first meet the requirement of relevant technical specification in terms of their use in asphalt mixture. The test results of basic technical property indexes of aggregate, filler and asphalt binder were listed in Table 2, Table 3 and Table 4, respectively. It can be seen from the results that all basic technical property indexes of raw materials meet the requirements of Chinese specification [32,33]. In detail, DGR was lighter than LP, and the density of the former was 6% lower than that of the latter. The gradation of DGR was slightly finer according to the particles size distribution shown in Table 3. The introduction of waste tire rubber powder has significantly lowered the penetration, ductility and raised the softening point of base asphalt binder. Table 4 also suggested that rubber powder gave asphalt binder excellent elasticity.

#### 3.1.2. Material Characteristics

##### Granite Aggregate

**Figure 5:**
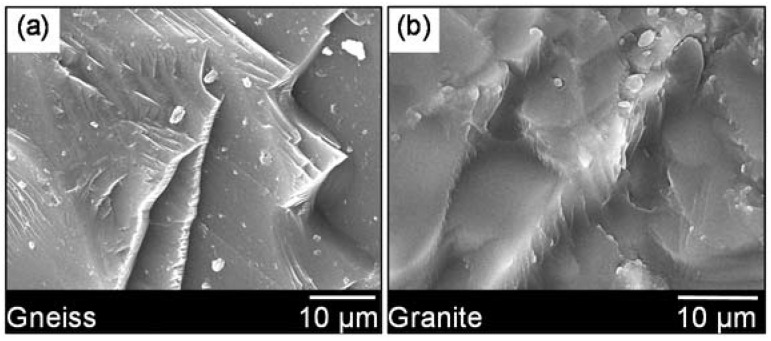
Micro-morphology images of aggregate particles: (**a**) Gneiss [15]; (**b**) Granite.

Micro-morphology and chemical composition of aggregates are very important features in terms of the use in asphalt mixture. The former affects the physical bonding behavior between aggregate and asphalt mastic, and the latter influences the chemical bonding behavior. Unlike the common acid gneiss aggregate with flat and layered surface texture (see picture a in Figure 5), the surface of granite was very coarse and spur-and-gully, as shown in Figure 5b. The rich surface feature was beneficial for the physical bonding behavior. Therefore, the poor chemical bonding behavior of aggregate and asphalt mastic should be responsible for the less-than-ideal performance of granite based asphalt mixture.

**Figure 6:**
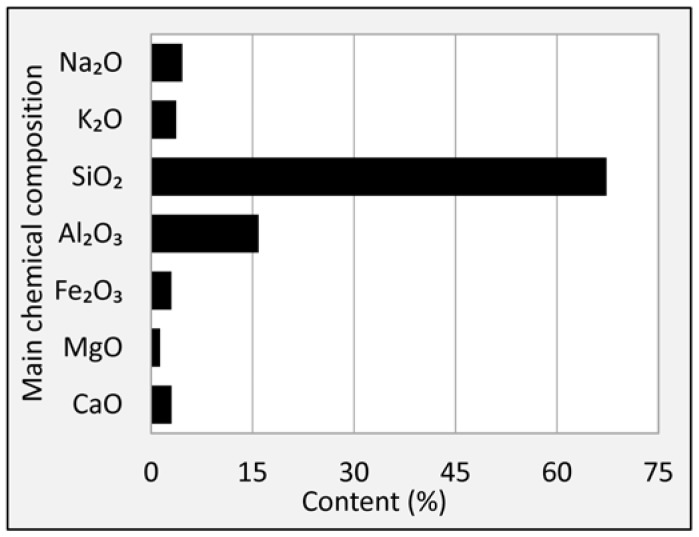
Main chemical compositions of granite.

The main chemical compositions of granite are shown in Figure 6. It can be seen that the SiO_2_ almost accounts for 70% by weight of granite. The acid–alkaline feature of rocks can be easily decided according to the content of SiO_2_ suggested by previous researchers, and the rock can be defined as acid aggregate when the content of SiO_2_ is beyond 65% [34]. Granite was actually a kind of typical acid aggregate according to this rule. High content of SiO_2_ resulted in poor chemical bonding of aggregate and asphalt mastic. Therefore, improving the bonding behavior of the granite mixture system by modifying other raw materials (filler and asphalt) was very important. Figure 6 also showed that granite contains more Al_2_O_3_ and some percent of Na_2_O, K_2_O, CaO, MgO and Fe_2_O_3_. This was related to the mineral phases of granite.

**Figure 7:**
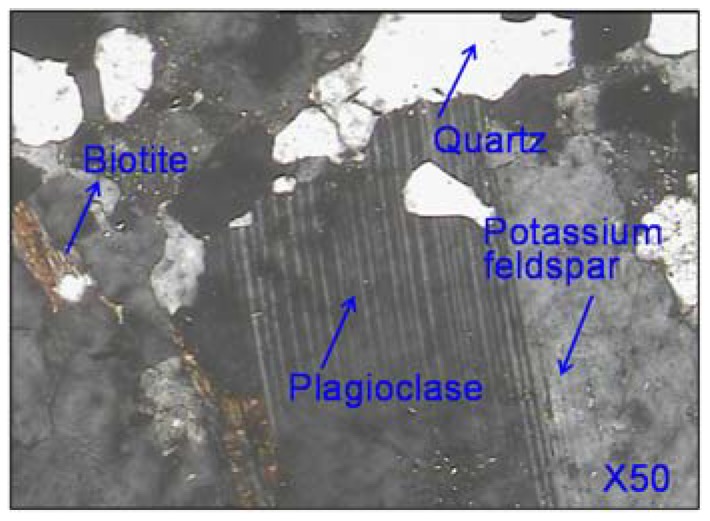
Mineral phases of granite.

The mineral phases of granite are shown in Figure 7. It suggested that quartz, plagioclase and potassium feldspar were the main mineral phases of granite, and small amount of biotite can be also observed. All these phases contain SiO_2_, as a result, the content of SiO_2_ in granite was very high. Quartz made the greatest contribution to the content of SiO_2_. All other phases also contain Al_2_O_3_ besides quartz, naturally, a large amount of Al_2_O_3_ was detected in granite as shown in Figure 6. The rest of Na_2_O, K_2_O, CaO, MgO and Fe_2_O_3_ mainly participated in the generation of plagioclase (Na(AlSi_3_O_8_), Ca(Al_2_Si_2_O_8_)), potassium feldspar (K_2_O·Al_2_O_3_·6SiO_2_) and biotite (K(Mg, Fe^2+^)_3_(Al,Fe^3+^)Si_3_O_10_(OH,F)_2_).

##### Fillers

**Figure 8:**
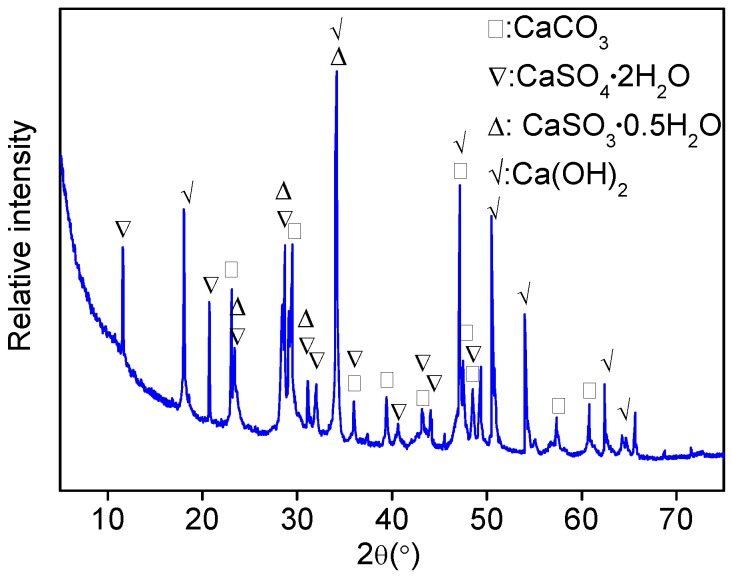
Mineral phases of DGR.

It is well known that the main phase of LP is calcium carbonate (CaCO_3_). The mineral phases of DGR are analyzed in this section. Figure 8 suggested that calcium carbonate, dihydrate gypsum (CaSO_4_·2H_2_O) and calcium hydroxide (Ca(OH)_2_) were the main phases of DGR. The phase types were directly related to the generation process of DGR. The absorbent for SO_2_ was a slurry of CaCO_3_/CaO, and CaO transformed into Ca(OH)_2_ under wet condition, which was the active ingredient for SO_2_ absorption. Ca(OH)_2_ turned into CaSO_3_·0.5H_2_O by absorbing SO_2_ in wet condition, which can be further oxidized into CaSO_4_·2H_2_O. It was the source of CaSO_4_·2H_2_O. This process was accompanied by the decomposition of CaCO_3_, which can provide sufficient CaO. It can be seen from Figure 8 that almost all strong diffraction peaks were possessed by Ca(OH)_2_. It meant that the dosage of CaCO_3_/CaO was excessive. A great amount of the remaining Ca(OH)_2_ gave DGR with high alkalinity, which was good for the bonding behavior of the asphalt mixture system.

The average chemical compositions of LP and DGR corresponded to the mineral phase results. The decomposition of CaCO_3_ in LP resulted in large amounts of CaO and loss on ignition (LoI). The mass ratio of CaO to LoI was 1.28, which was very close to the molecular weight ratio of CaO to CO_2_. It proved that the release of CO_2_ should be responsible for the large LoI in LP. For DGR, the contents of CaO, SO_3_ and LoI were much larger than that of other chemical compositions. The CaO was related to CaCO_3_, Ca(OH)_2_, CaSO_3_·0.5H_2_O and CaSO_4_·2H_2_O phase. Supposing the large LoI was completely caused by the decomposition of CaCO_3_ (in fact, the dehydration process of mineral phases also made contribution to it), the amount of CaO generated during the release of CO_2_ was 18.98%. The content of CaO corresponding to CaSO_3_·0.5H_2_O and CaSO_4_·2H_2_O was 14.15%, computed according to the total content of SO_3_ shown in Table 5. The total content of CaO for DGR was 59.88%, therefore, there were about 26.75% of CaO was from Ca(OH)_2_. It also indicated that the amount of retaining Ca(OH)_2_ was large, which contributed to the alkaline of DGR.

**Figure 9:**
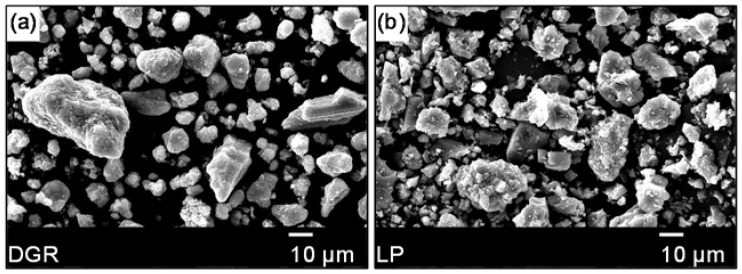
Micro-morphology images of fillers: (**a**) DGR, (**b**) LP.

The micro-morphology images of DGR and LP are shown in Figure 9. The figures suggested that the surface texture of commonly used LP filler particles was coarser than that of DGR particles. Furthermore, the outline of DGR particles was much sleeker and more ellipsoid than that of common used LP filler. Ellipsoid particles could make the asphalt mastic easy to flow at high temperature because of poor friction action between particles. This disadvantage may harm the pavement performance of asphalt mixture, especially the high-temperature stability. The specific situation will be revealed in the section of pavement performance evaluation.

**Figure 10:**
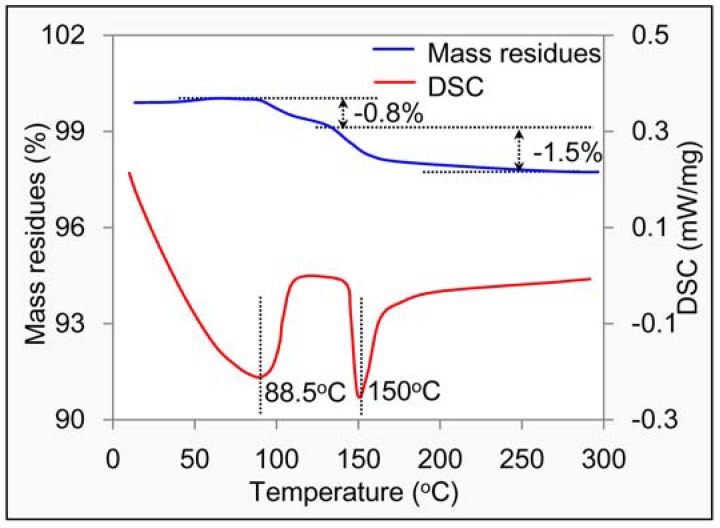
TG-DSC analysis result of DGR.

Asphalt mixture was prepared at a high temperature of 165 °C for base asphalt and 185 °C for modified asphalt. Therefore, evaluating the thermal stability of DGR as a type of new filler was very important. Figure 10 shows the thermal analysis result of DGR. It shows that there are two stages. In the first stage, a strong endothermic peak appeared at 88.5 °C due to the volatilization of free water contained in DGR. It accordingly resulted in 0.8% of mass loss in this stage. In the second stage, another endothermic peak appeared at 150 °C; it belonged to the crystal water release of CaSO_4_·2H_2_O, and dihydrate gypsum transformed into half-water gypsum during this stage. About 1.5% of mass loss was seen. The total mass reduction was quite small, hence, the thermal stability of DGR was good in terms of mass change, while the phase types changed at the mixing temperature of asphalt mixture.

##### Asphalt Binders

**Figure 11:**
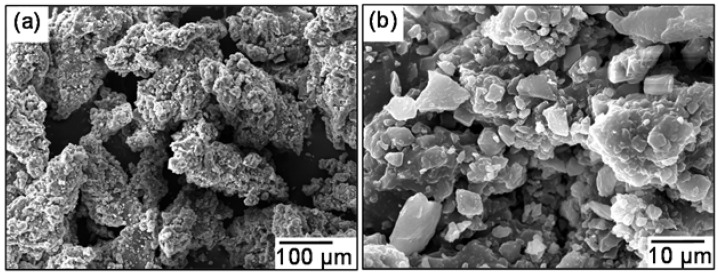
Micro-morphology images of crumb rubber particles: (**a**) under low magnification times; (**b**) under high magnification times.

The micro-morphology images of used rubber particles are displayed in Figure 11. Picture a showed that the edges and corners of the rubber particles were quite clear, and their appearance was blistered. The large amount of free fine particles and rich channel structures contributed to the blistered surface. The coarse and blistered surface could result in the adsorption and absorption of asphalt, which was a benefit for the compatibility and integrity of base asphalt and crumb rubber particles. Using crumb rubber to modify asphalt was advantageous in terms of its micro-morphology feature.

**Figure 12:**
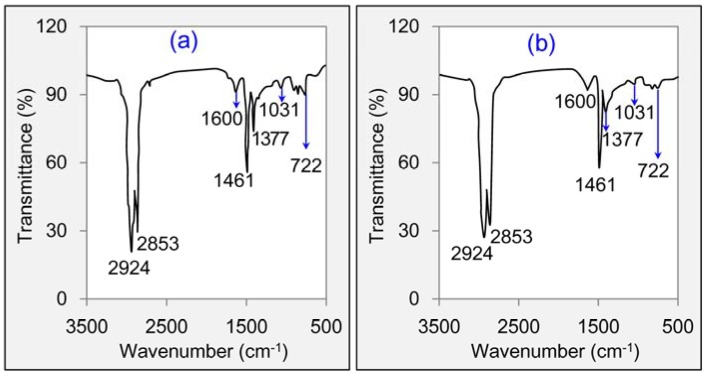
FT-IR analysis results: (**a**) Base asphalt, (**b**) RMA.

Figure 12 gave the functional group analysis results of base asphalt and RMA. It can be seen that the infrared spectrum curves of these two asphalts were similar. The main characteristic peaks were displayed at wavenumbers of 2924, 2853, 1600, 1461, 1377, 1031 and 722 cm^−1^. The peaks at 2924 and 2853 cm^−1^ attributed to the stretching vibration of –CH_2_– bond and C–H bond. And the peaks at 1600, 1461 and 1377 cm^−1^ attributed to the stretching vibration of C=C bond, in-plane bending vibration of –CH_2_– and bending vibration of C–H. The infrared spectrum curves and peak strengths of base asphalt and RMA at wavenumbers of 500–1400 cm^−1^ was a little different. The characteristic peak strengths of RMA such as at 1377, 1031 and 722 cm^−1^ were smaller than that of base asphalt. It suggested that the introduction of crumb rubber may change the structural network of asphalt binder slightly. While the effect was limited. Conclusions can be made that mainly have physical changes during the mixing of crumb rubber and base asphalt.

### 3.2. Asphalt Mixture and Its Pavement Performances

#### 3.2.1. Design Results of Asphalt Mixtures

The volumetric properties of four asphalt mixtures used in this research are shown in Table 6. It can be seen from the results that all volumetric indexes met the design requirements. The optimum asphalt content (OAC) of asphalt mixture containing RMA was higher than that of asphalt mixture with base asphalt. The increment of asphalt content caused by RMA was 0.4%. This was mainly due to the reduction of base asphalt content in RMA. More RMA was needed in order to fully wet the mineral mixture. The OAC values of asphalt mixtures containing different fillers were the same when the same type of asphalt binder was used. It indicated that the effect of filler types on the asphalt content was weak. Table 6 also shows that the air void and void in mineral aggregate (VMA) of asphalt mixture containing DGR were slightly lower than that of asphalt mixture with LP. It suggested that DGR made the asphalt mixture a little densely compacted. The VMAs of asphalt mixtures prepared with RMA were obviously higher than that of asphalt mixture with base asphalt. It meant the RMA lowered the compatibility of asphalt mixture.

#### 3.2.2. Moisture-Induced Damage Resistance

**Figure 13:**
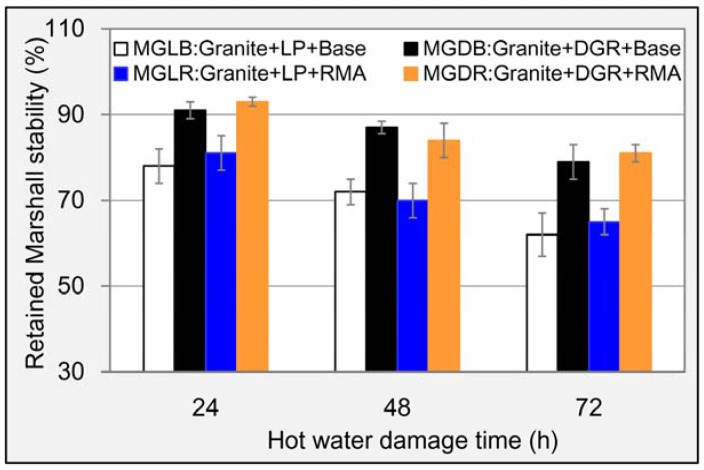
RMS results of different asphalt mixtures.

The original Marshall stabilities of M_GLB_, M_GDB_, M_GLR_ and M_GDR_ were 8.4, 8.9, 11.5 and 10.9 kN, respectively. This suggested that the contribution of RMA on increasing Marshall stability was big, and the increments for LP and DGR asphalt mixtures were 3.1 and 2.0 kN, respectively, while the effect of DGR on Marshall stability was weak and inconsistent for asphalt mixtures with different asphalt. The RMS values for each mixture after suffering different times of hot water damage are listed in Figure 13. The downward trend for all asphalt mixtures is displayed with the rise of damage time. Hence, the damage caused by hot water was serious. DGR showed a very positive role in maintaining the stability of asphalt mixture according to the results. For base asphalt, the RMS of asphalt mixture prepared with DGR was still up to 79% even after hot water damage for 72 h, and the value for LP asphalt mixture was only 62% under the same condition. For RMA, RMS of DGR asphalt mixture was as high as 81% after damage for 72 h, which was 16% numerically larger than that of LP asphalt mixture. Figure 13 also revealed that the type of asphalt weakly effected hot-water stability. The RMS of asphalt mixture containing LP and DGR simultaneously was largest among these four asphalt mixtures after a longtime hot water damage. Hence, it is preferable to prepare asphalt mixture with LP and DGR considering the hot water damage resistance.

**Figure 14:**
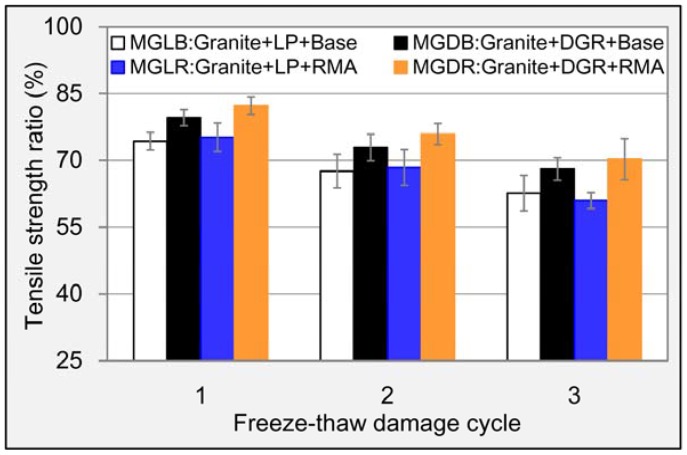
TSR results of different asphalt mixtures.

The original splitting strengths of M_GLB_, M_GDB_, M_GLR_ and M_GDR_ were 0.94, 0.97, 1.05 and 1.10 MPa, respectively. Hence, DGR and RMA both can enhance the strength of asphalt mixture. Compared to LP, DGR improved the strength of base asphalt mixture and RMA mixture by 3.2% and 4.8%, respectively. For RMA, the improved percentages were 11.7% and 13.4% for LP asphalt mixture and DGR asphalt mixture, respectively, compared to base asphalt. So the contribution made by RMA was much more obvious. Similar to the change rule of RMS, TSR for each mixture decreased gradually with the rise of freeze-thaw damage cycles (see Figure 14). DGR also performed better than RMA in maintaining the strength of asphalt mixture. Furthermore, compared to LP, DGR raised TSR value of base asphalt mixture and RMA mixture by 5.4% and 9.3%, respectively, even after three cycle’s freeze-thaw damage. For RMA, the situation was a little complex. RMA did better than base asphalt in maintain the strength of LP asphalt mixture at early stage of freeze-thaw damage. The rule gradually turned to the opposite with the strengthening of the damage degree. It meant that the durability of bonding feature between aggregate and RMA was insufficient. The strength loss of asphalt mixture containing RMA and DGR was smallest among these asphalt mixtures even serious freeze-thaw damage for three cycles was applied. It meant that the combination of RMA and DGR was also a good method to improve the freeze-thaw damage resistance ability.

**Figure 15:**
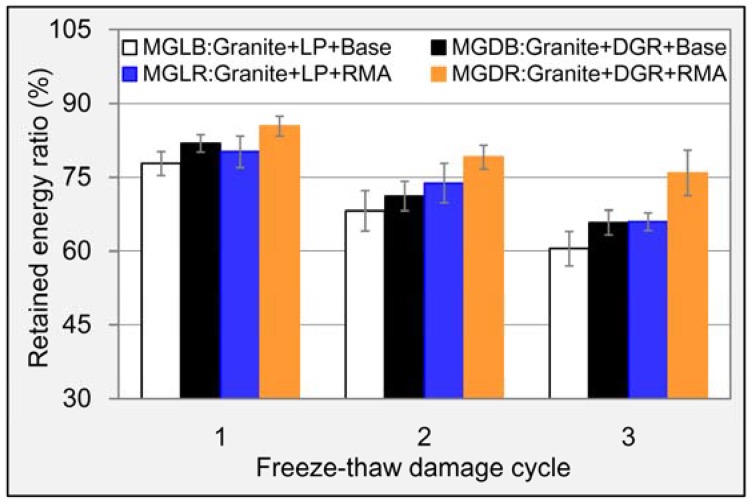
RER results of different asphalt mixtures.

Figure 15 gave the computed results of RER. It can be seen that the change rule of RER was a little different from that of TSR. RMA also displayed an obvious effect on RER. Compared to base asphalt, the increment of RER caused by RMA were 5.5% and 10.1% for LP asphalt mixture and DGR asphalt mixture, respectively, even after three cycle’s freeze-thaw damage. For DGR, its contribution were 5.3% and 9.9% for base asphalt mixture and RMA mixture, respectively. And the RER of asphalt mixture containing DGR and RMA was still up to 75.9% after serious freeze-thaw damage. The loss ratio of RER was less than that of spitting strength; this was because of the crumb rubber contained in RMA which enhanced the flexibility and the ability of bearing big deformation. Large deformation will result in big fracture energy according to Equation (3). Concluding this section, the combined use of DGR and RMA played a much more positive role in improving the water damage resistance of asphalt mixture than when they were used alone.

#### 3.2.3. Low-Temperature Crack Resistance

**Figure 16:**
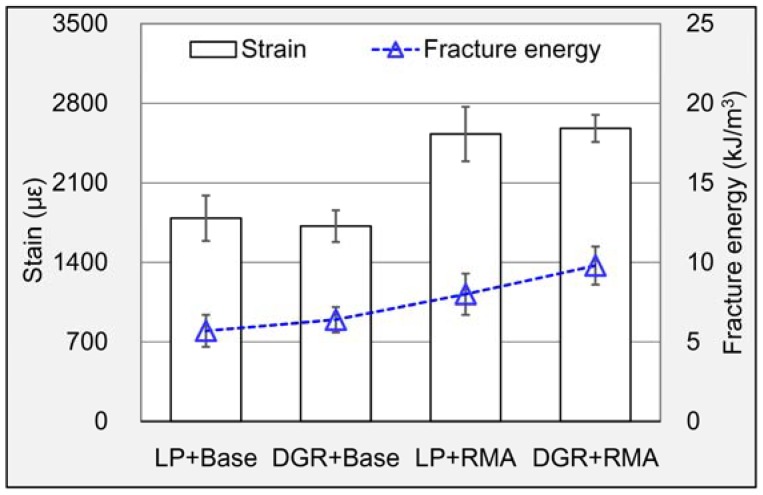
Strain and fracture energy of different asphalt mixtures.

The computed strain and fracture energy of these four asphalt mixtures are shown in Figure 16. In terms of strain, LP and DGR almost performed the same either in base asphalt mixture or RMA mixture. While the effect of RMA on the low-temperature strain was significant. Compared to base asphalt, RMA increased the strain of LP asphalt mixture and RMA mixture by 41.3% and 50.0%, respectively. It indicated that the introduction of crumb rubber enhanced the deformability of asphalt mixture at low temperature. In terms of fracture energy, the energy values were gradually rising by changing filler types and asphalt types in turn. It suggested that the DGR and RMA both can improve the fracture energy of asphalt mixture. Asphalt mixture prepared with DGR and RMA possessed the largest fracture energy, about 10 kJ/m^3^. Therefore, similar to moisture stability, the combination of DGR and RMA did better in improving the low-temperature performance of asphalt mixture.

#### 3.2.4. High-Temperature Deformation Resistance

**Figure 17:**
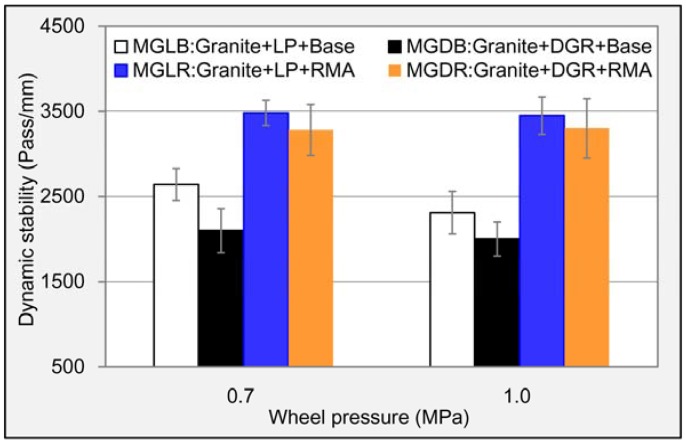
Dynamic stability results of different asphalt mixtures.

The high-temperature stability is very important to the durability of asphalt pavement especially at some regions of higher temperature. The computed dynamic stabilities of the four asphalt mixtures are displayed in Figure 17. It clearly shows that the DGR worsened the high-temperature deformation resistance of asphalt mixture. Specifically, at wheel pressure of 0.7 MPa, the reduction percent of dynamic stabilities for base asphalt mixture and RMA mixture caused by DGR were 20.5% and 5.7%, respectively, and which were 13.4% and 4.3% at wheel pressure of 1.0 MPa. This may be due to the sleek and ellipsoid appearance of DGR particles, which made asphalt mastic easy to flow. The improvement made by the rubber was compelling. At wheel pressure of 0.7 MPa, 31.8% and 56.2% higher dynamic stability were shown for LP asphalt mixture and DGR asphalt mixture, respectively, compared to base asphalt. The values were 49.4% and 65.0%, respectively, at wheel pressure of 1.0 MPa. This was mainly due to the contribution of rubber’s excellent elasticity. The results suggested that RMA obviously made up the weakness of DGR in terms of high-temperature performance of asphalt mixture. Figure 17 also indicated that the change of dynamic stability for RMA mixture was quite small when applied wheel pressure increasing from 0.7 MPa to 1.0 MPa. Hence, the RMA was suitable for the road located in the high-temperature region.

#### 3.2.5. Fatigue Crack Resistance

**Figure 18:**
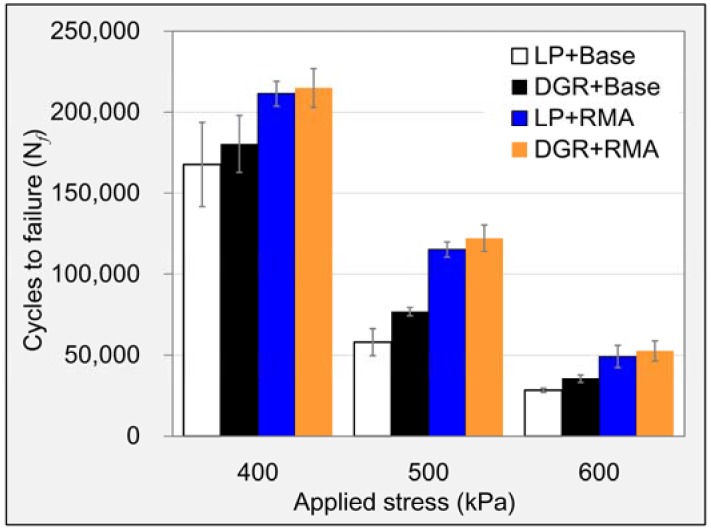
Fatigue life test results of different asphalt mixtures.

Figure 18 gave the fatigue life results of every asphalt mixture at each stress level. It displayed that the fatigue lives of all asphalt mixtures rapidly decreased with the rising of stress levels. A more significant fluctuation can be observed at the low stress level. DGR and RMA both showed good potential to raise fatigue life of asphalt mixture. Furthermore, DGR raised the fatigue lives of base asphalt mixtures tested at 400, 500 and 600 kPa by 7.6%, 32.4% and 25.0%, respectively. The values were 1.7%, 6.0% and 7.1% for RMA mixture. In terms of RMA, it increased the fatigue lives of LP asphalt mixtures tested at 400, 500 and 600 kPa by 26.1%, 98.6% and 73.5%, respectively, and the percentage increases in fatigue lives were 19.2%, 58.9%, and 48.6%, respectively, for DGR asphalt mixtures. It indicated that the improvement of fatigue life made by RMA was much more obvious. Figure 18 also showed that asphalt mixture containing DGR and RMA simultaneously always possessed the highest fatigue lives among the four asphalt mixtures at all stress levels. So the combination of DGR and RMA also did better in improving fatigue life of asphalt mixture than when they were used alone.

The relationship between fatigue life, applied stress and initial strain is commonly expressed as Equations (6) and (7), respectively. The parameters (*L*, *m*, *K*, *n*) contained in the fatigue equations were determined by regression analysis of obtained test raw data. Three replicates for every asphalt mixture at each stress level were applied in this research. The data points would be highly overlapped in a coordinate system with the stress level as abscissa if the fatigue performance was analyzed by means of Equation (6). Considering this, Equation (7) was used in this research. The fatigue life and initial strain should meet a linear relationship at a double logarithmic coordinate system according to Equation (7).

(6) $$N_{f}=L{\left(\frac{1}{\sigma_{0}}\right)}^{m}$$

(7) $$N_{f}=K{\left(\varepsilon_{0}\right)}^{n}$$

where *N_f_* is the fatigue life (loading cycles to failure), *σ*_0_ is applied stress level, ε_0_ is the initial strain of sample (the strain caused by applied stress level), and *L*, *K*, *m*, *n* are material constants of the tested asphalt mixture.

**Figure 19:**
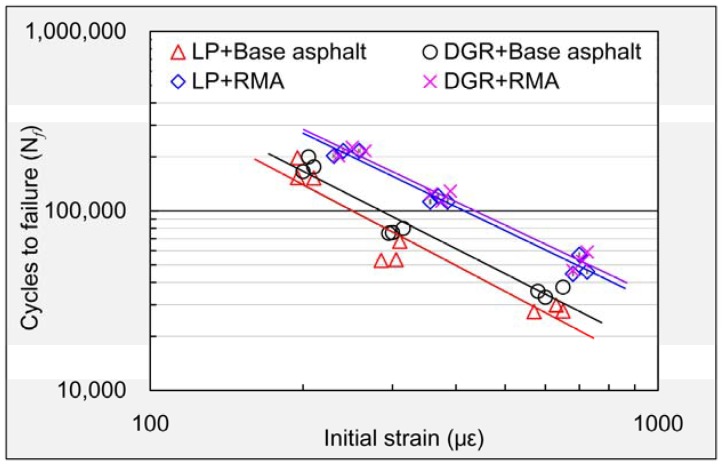
Regression analysis of obtained data points of fatigue life.

The relationship between fatigue life and initial strain caused by applied stress was displayed in a double logarithmic coordinate system as shown in Figure 19. The fatigue life and initial strain should meet linear relationship at double logarithmic coordinate system according to Equation (7). The parameter *n* in Equation (7) corresponded to the slope of the fitting line shown in Figure 19. So the absolute value of parameter *n* can reflect the falling speed of fatigue life for certain asphalt mixture with the increase of stress level. It can be seen from the figure that the absolute values of parameter *n* for M_GLB_, M_GDB_, M_GLR_ and M_GDR_ were gradually reduced. It meant that the fatigue durability of asphalt mixture prepared with DGR and RMA was the best among these asphalt mixtures, and the fatigue equations determined by regression analysis (see Table 7) also supported this conclusion, although the absolute value of parameter *n* (1.34) was just a little smaller than that of asphalt mixture containing LP and RMA (1.36).

## 4. Conclusions

In terms of raw material characteristics and main pavement performances of asphalt mixture, the feasibility of preparing asphalt mixture by means of low-grade granite aggregate, rubber modified asphalt (RMA) and desulphurization gypsum residues (DGR) simultaneously was investigated in this research. The basic technical properties and main material characteristics of raw materials were first detected in order to fully recognize them. Then the pavement performances of asphalt mixture prepared with granite aggregate, DGR and RMA were fully evaluated to reveal the feasibility. Based on the research results, the following conclusions can be drawn.

(1) Unlike the common acid gneiss aggregate with flat and layered surface texture, the surface of granite was very coarse and spur-and-gully. The quartz, plagioclase and potassium feldspar were the main mineral phases of granite, and they were responsible for the high content of SiO_2_ in granite;
(2) The large amount of Ca(OH)_2_ from the excess absorbent of SO_2_ increased the percent of alkaline ingredients in DGR, which made DGR an potential modifier to enhance the bonding property of asphalt mixture system. The ellipsoid outline of DGR particles would increase the liquidity of mastic theoretically, which was not good for the high-temperature stability of asphalt mixture;
(3) DGR made asphalt mixture easier to be compacted. RMA was just the opposite, it lowered the compatibility of asphalt mixture. The used content of RMA was larger than that of base asphalt binder, and this was due to the reduction of base asphalt percentage in RMA. Much more RMA was needed in order to fully wet the surface of mineral particles;
(4) DGR showed a very positive role in strengthening the moisture-induced damage resistance, and the combination of DGR and RMA even did better although the effect of RMA on the moisture stability of granite asphalt mixture was slight and erratic;
(5) DGR significantly worsened the high-temperature deformation resistance of granite asphalt mixture, and the improvement of the high-temperature stability made by RMA was compelling. The combination of RMA and DGR can make up the weakness of DGR in terms of the high-temperature performance of granite asphalt mixture;
(6) DGR and RMA both showed an excellent ability to increase low-temperature crack resistance, fatigue life and the fatigue durability of granite asphalt mixture. The combined use of DGR and RMA in the granite asphalt mixture also performed much better than when they were used alone.

## Figures and Tables

**Table 1:** Combination of raw materials types for each mixture.

Mixtures	Coarse Aggregate	Fine Aggregate	Filler	Asphalt
M_GLB_	Granite	Granite	LP	Base asphalt
M_GDB_	Granite	Granite	DGR	Base asphalt
M_GLR_	Granite	Granite	LP	RMA
M_GDR_	Granite	Granite	DGR	RMA

**Table 2:** Basic technical properties of granite aggregates.

Measured Index	Coarse Aggregate			Fine Aggregate	Requirements in China
Size Range (mm)	19–9.5	9.5–4.75	4.75–2.36	2.36–0	
Apparent specific gravity	2.719	2.722	2.717	2.701	≥2.5
Water absorption (%)	0.4	0.6	0.6	1.1	≤2
Flakiness and elongation (%)	7.9	10.8	NA	NA	≤18
Los Angeles abrasion (%)	23.8	23.8	21.4	NA	≤30
Crush value (%)	19.9	NA	NA	NA	≤28
Fine aggregate angularity (%)	NA	NA	NA	51	≥30
Sand equivalent (%)	NA	NA	NA	66	≥60

**Note.** “NA” stands for the index is not applicable at that situation, and the same in latter tables.

**Table 3:** Basic technical properties of fillers.

Measured Index		LP	DGR	Requirements in China
Specific gravity (g/cm^3^)		2.728	2.557	≥2.5
Percent passing (%)	0.6 mm	100	100	100
	0.15 mm	95.2	96.1	90–100
	0.075 mm	87.1	90.3	75–100

**Table 4:** Basic technical properties of base asphalt and RMA.

Measured Index	Base Asphalt	RMA	Requirements in China	
			Base Asphalt	RMA
Penetration (25 °C; 0.1 mm)	68	46	60–80	30–60
Ductility (base asphalt, 15 °C; RMA, 5 °C; cm)	155	8.9	≥100	≥5
Softening point (°C)	47.2	70.1	≥46	≥60
Elasticity resume (25 °C; %)	NA	79	NA	≥60

**Table 5:** Average chemical compositions of DGR and LP.

Filler	Content (%)								
	SiO_2_	Al_2_O_3_	CaO	Fe_2_O_3_	MgO	K_2_O	SO_3_	LoI	Others
DGR	0.41	0.22	59.88	0.61	1.12	0.67	20.21	14.91	1.97
LP	3.01	1.01	53.02	0.54	0.28	0.22	0.06	41.35	0.51

**Table 6:** Volumetric properties of designed asphalt mixtures.

Volumetric Property	Mixture Type				Design Requirement
	M_GLB_	M_GDB_	M_GLR_	M_GDR_	
Optimum asphalt content (%)	5.0	5.0	5.4	5.4	NA
Air voids (%)	4.5	4.3	4.7	4.5	4–6
Voids in mineral aggregate (%)	14.1	13.8	15.4	15.1	≥13
Voids filled with asphalt (%)	69.5	68.8	69.5	70.2	65–75

**Table 7:** Fatigue equations of different asphalt mixtures.

Mixture	Parameters		Fatigue Equation	Correlation Coefficient
	*K*	*n*		R^2^
M_GLB_	3.7 × 10^8^	−1.49	*N_f_* = 3.7 × 10^8^(ε_0_)^−1.49^	0.95
M_GDB_	3.3 × 10^8^	−1.44	*N_f_* = 3.7 × 10^8^(ε_0_)^−1.44^	0.90
M_GLR_	3.7 × 10^8^	−1.36	*N_f_* = 3.7 × 10^8^(ε_0_)^−1.36^	0.97
M_GDR_	3.4 × 10^8^	−1.34	*N_f_* = 3.7 × 10^8^(ε_0_)^−1.34^	0.98
